# Malignant Gastric Outlet Obstruction Caused by Duodenal Cervix Metastasis in a Young Woman: Rendezvous Technique

**DOI:** 10.3390/medicina57080765

**Published:** 2021-07-28

**Authors:** Ester Marra, Pasquale Quassone, Pasquale Tammaro, Cinzia Cardalesi, Raffaele D’Avino, Fabio Cipolletta, Anna Del Prete, Angela Travaglino, Stefania Tamburrini, Giovanni Ferrandino, Giuseppe Sarti, Michele Iannuzzi, Pietro Maida, Gianpaolo Santini

**Affiliations:** 1 Department of Surgery, University of Naples Federico II, 80138 Naples, Italy; estermarra9@gmail.com; 2 Department of Radiology, Università degli Studi della Campania Luigi Vanvitelli, 80138 Naples, Italy; pasquale.quassone@gmail.com; 3 Department of Surgery, Ospedale del Mare, ASL NA1 Centro, 80147 Naples, Italy; pasqualetammaro81@gmail.com (P.T.); raffaele.davino80@gmail.com (R.D.); angelatravaglino@gmail.com (A.T.); p.maida@libero.it (P.M.); 4 Department of Oncology, Ospedale del Mare, ASL NA1 Centro, 80147 Naples, Italy; cinzia.cardalesi@gmail.com; 5 Department of Gastroenterology, Ospedale del Mare, ASL NA1 Centro, 80147 Naples, Italy; cipolletta.f@gmail.com (F.C.); annadelprete84@libero.it (A.D.P.); 6 Department of Radiology, Ospedale del Mare, ASL NA1 Centro, 80147 Naples, Italy; gianni.ferrandino90@gmail.com (G.F.); sartigiuseppe@gmail.com (G.S.); santinigianpaolo@libero.it (G.S.); 7 Department of Anesthesiology and Intensive Care, Ospedale del Mare, ASL NA1 Centro, 80127 Naples, Italy; michele.iannuzzi74@gmail.com

**Keywords:** duodenal obstruction, SEMSs, rendezvous procedure

## Abstract

*Background*: Malignant gastric outlet obstruction (MGOD) is an extremely rare expression of advanced extra-gastrointestinal cancer, such as squamous cell carcinoma (SCC) of the cervix, and only sixcases are described in the literature.Because of the short life expectancyand the high surgical risk involving these patients, less invasive approaches have been developed over time, such asthe use of an enteral stent or less invasive surgical techniques (i.e., laparoscopic gastrojejunostomy). However, MGOD could make it difficult to perform an endoscopic retrograde cholangio-pancreatography (ERCP) for standard endoscopic drainage, so in this case a combined endoscopic-percutaneous technique may be performed. This article, therefore, aims to highlight the presence in the doctor’s armamentarium of the “rendezvous technique”, few case reports of whichare described in the literature, and, moreover, this article aims to underline the technique’sfeasibility. Case Presentation: The case is that of a 38-year-old woman who presented with MGOD three years after the diagnosis of SCC of the cervix, who successfully underwent the rendezvous technique with the resolution of duodenal obstruction. Endoscopic enteral stenting treatment with the placement of a metal stent (SEMSs) represents the mainstay of MGOD treatment compared withsurgery due to its lower morbidity, mortality, shorter hospitalization and earlier symptom relief. However, in patients with both duodenal and biliary obstruction, a combined endoscopic–percutaneous approach may be necessary because of the difficulty in passing the duodenal stricture or in accessing the papilla through the mesh of the duodenal SEMS. *Conclusion*: The rendezvous procedure is a technicallyfeasible and minimally invasive approach to the double stenting of biliary and duodenal strictures. It achieves the desired therapeutic result while avoiding the need to perform more invasive procedures that could have a negative impact on the patient’sprognosis.

## 1. Introduction

Malignant gastric outlet obstruction (MGOD) refers toa mechanical obstruction of the pylorus or the duodenum due to compression/infiltration from advanced loco-regional malignancies [1,2]. Less common causes of MGOD are extra-gastrointestinal cancer metastasis, such as melanoma or squamous cell carcinoma (SCC) of the cervix [1,3]. Gastrointestinal involvement caused by cervicalcancer metastasis occurs in approximately 8% of patients in the recto-sigmoid tract as a result of local extension;gastric metastatic lesions are identified in fewerthan 2% of patients, and isolated metastases to the small bowel are exceedingly rare [4].

Patients typically present with nausea and vomiting, and they are often unable to take liquids or solid food with consequent malnutrition, dehydration and weight loss. Endoscopic enteral stentingtreatment with placement of a metal stent(SEMSs) represents the mainstay of treatment compared withsurgery due to its lower morbidity, mortality, shorter hospitalization and earlier symptom relief [5,6,7,8]. However, a stent’s occlusion due to food impaction or tumor progression and its growth through thestent’s mesh can cause the recurrence of gastric obstruction symptoms and the need forendoscopic re-intervention. In the case of re-intervention and thefailure to pass through the duodenal stricture or to access the papilla through the mesh of the duodenal SEMS, a percutaneous approach may be necessary to overcome the obstruction [8,9] According to SCARE guidelines(Surgical CAseREport guidelines) [10], here is presented the case of a 38-year-old woman with a medical history of cervicalcarcinoma who was referred to hospital for serrated duodenal stricture caused by a cervicalmetastasis managed with a combined endoscopic–percutaneous rendezvous technique.

## 2. Case Discussion

**Figure 1:**
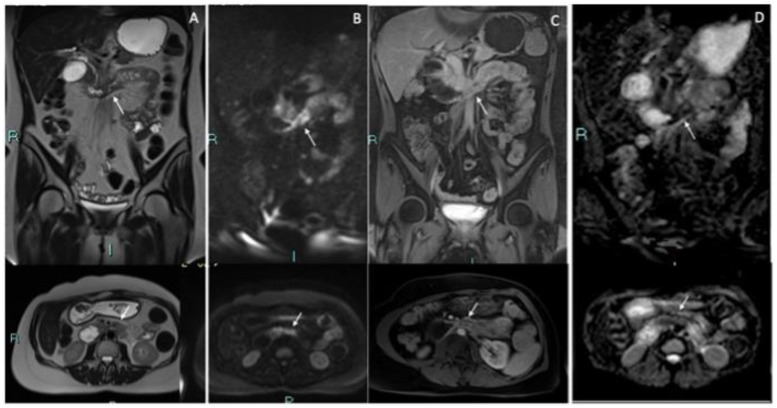
Pre-operative MRI (Magnetic Resonance Imaging) examination was per Figure 2. w-haste images and axial T2w-haste images showed severe stenosis of the third duodenal portion. (**B**,**C**) DWI (Diffusion Weighted Imaging) (**A**) and ADC (Apparent Diffusion Coefficient) (**B**) sequences coronal and axial images showed an evident restriction of diffusivity due to the marked increase in cellularity in the neoplastic tissue (**D**). Coronal and axial T1 and post-contrast (gadolinium) images showed marked contrast impregnation of the wall. There was no significant biliary dilatation.

**Figure 2:**
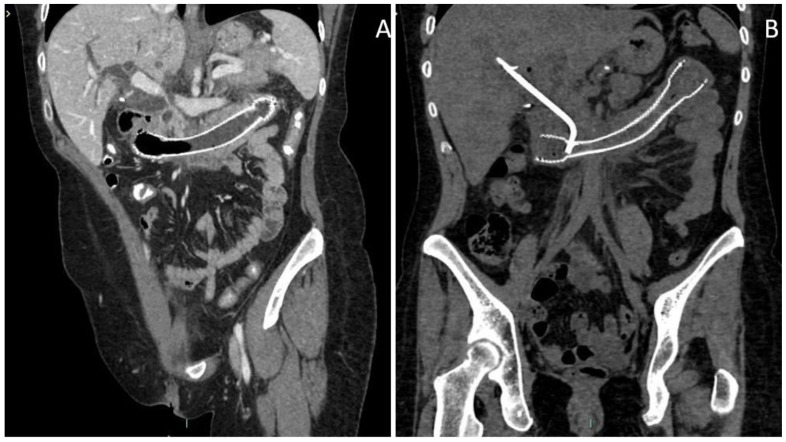
(**A**) Oblique MPR (Multi Planar Reconstruction) CT (Computed Tomography) image after duodenal stent placement, showing a severe dilation of the biliary tree, not present in the previous MRI exam, involving the common biliary duct. The course of the common biliary tract was grossly interrupted at the terminal end by compression of the papillary area due to the neoplastic infiltration of the stent mesh. (**B**) Oblique MPR CT image after biliary stent placement with the rendezvous approach due to the impossibility of reaching the papilla endoscopically due to tumor growth through duodenal stent’s mesh. Resolution of biliary dilatation is documented.

**Figure 3:**
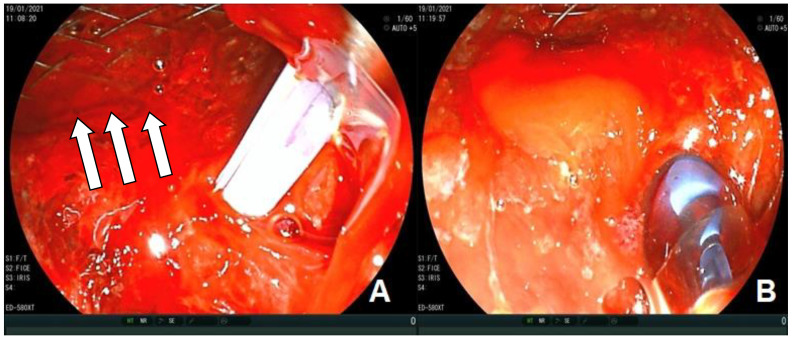
(**A**,**B**) ERCP (Endoscopic Retrograde Cholangio-Pancreatography)examination. Neoplastic infiltration (arrows) through duodenal mesh with consequent detection of stent obstruction.

**Figure 4:**
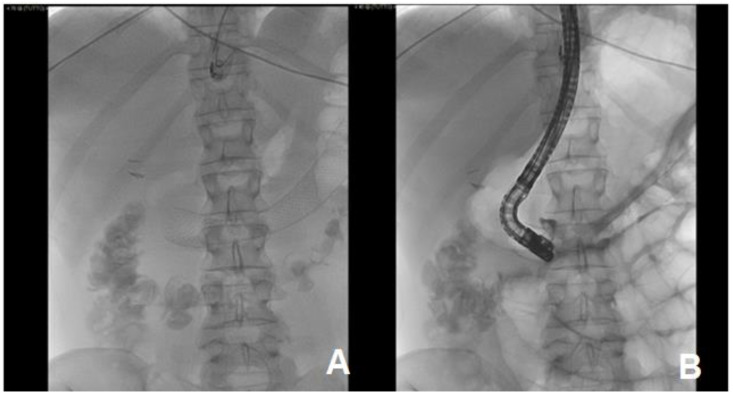
PTC (percutaneous transhepatic cholangiography). (**A**) On the left, imaging of the upper abdomen with evidence of a stent in duodenum; (**B**) on the right, the sphincterotome reaches the duodenal papilla, which appears impassable.

**Figure 5:**
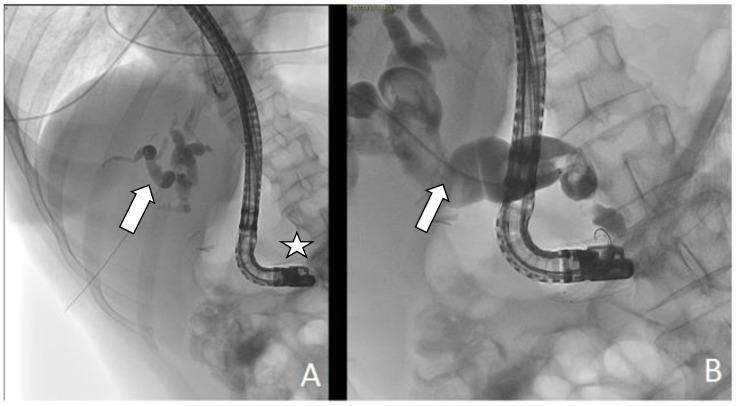
PTC. (**A**) On the left, the image shows the execution of a PTC (percutaneous transhepatic cholangiography) showing some biliary branches (arrow), while the sphincterotome is stationary at the level of major papilla (star). (**B**) On the right, the catheter (arrow) was inserted through percutaneous access and reached the major papilla.

**Figure 6:**
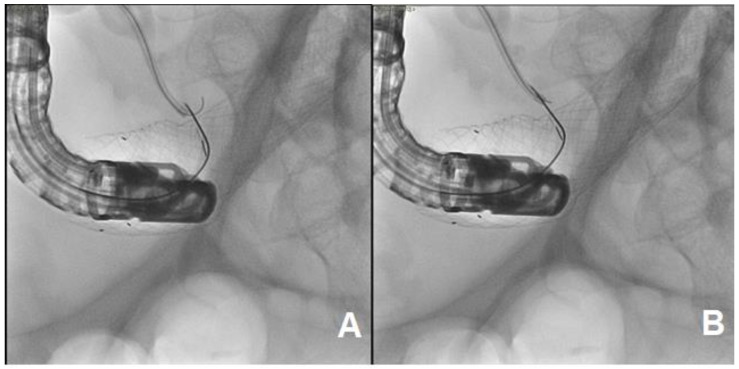
PTC. (**A**) On the left, the Teflon-coated guidewire that goes beyond the major papilla. (**B**) On the right, a guidewire was inserted through the sphincterotome and reached the main bile duct, overcoming the major papilla.

**Figure 7:**
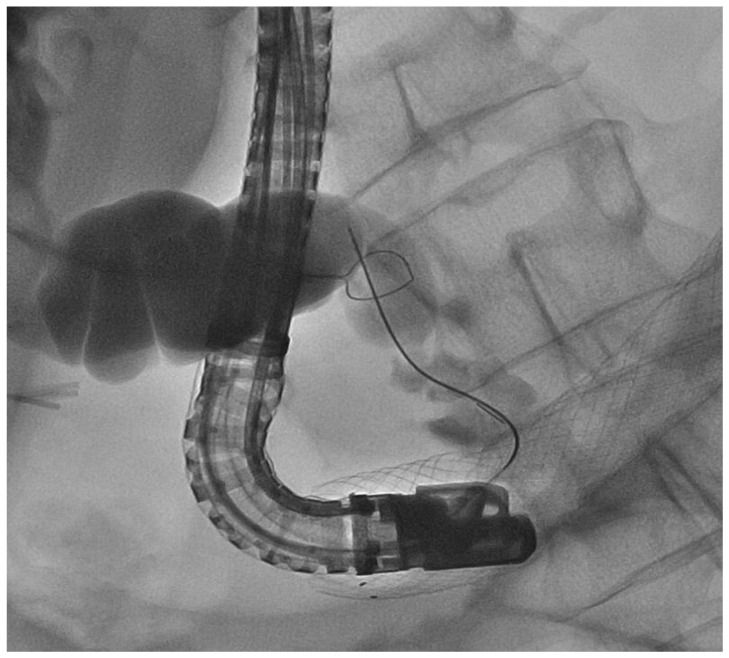
In this PTC image, the guidewire of the sphincterotome in the main bile duct is appreciable, while the One-Snare retrieval device, through the already present percutaneous catheter, was brought up to the guidewire itself and hooked it.

**Figure 8:**
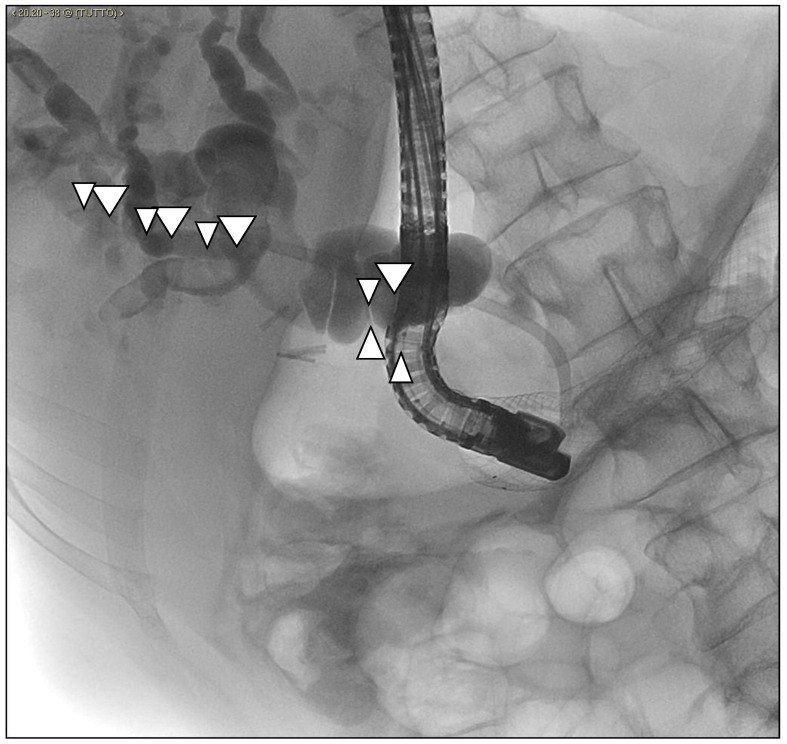
An angioplasty balloon (arrows) is mounted on the guidewire previously brought out by percutaneous retrograde (through the One-Snare device), which here appears already inflated, capable of breaking the stent links and widening the greater papilla-inflated balloon through the biliary duct and the major papilla.

**Figure 9:**
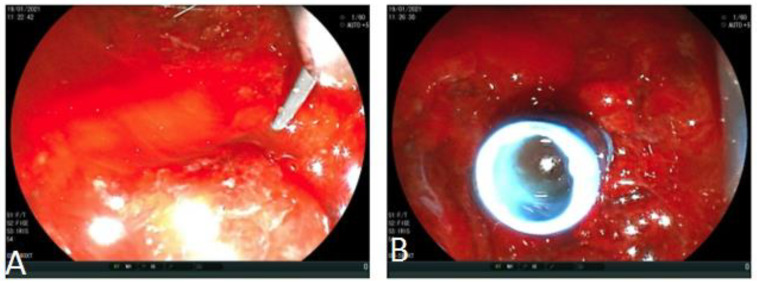
ERCP (**A**,**B**). insertion of a plasticbiliary prosthesis 9 cm 10 Fr through metallic duodenal mesh with resolution of obstruction.

A 38-year-old woman was referred to hospital to resolve a serrated duodenal stricture caused by a SCC cervicalmetastasis. She underwent hysterectomy, adjuvant chemotherapy (bevacizumab + taxolo + cisplatino) and radiotherapy. Because of the advanced stage of the disease (stage IV) (Figure 1), an endoscopic retrograde cholangio-pancreatography(ERCP) was performed in order to place an uncovered self-expandable metallic stent (SEMS) (12 cm length, 22 diameter) to resolve the obstruction. The post-procedure course was complicated by pancreatitis, severe jaundice and cholestasis, with an increase of pancreatic enzymes (amylase 550 U/I and lipase 370 U/L), alteration of liver function tests (total bilirubin 4.44 mg/dL, AST 578 mg/dL and ALT 938 mg/dL) and associated severe abdominal pain. Abdominal computed tomography (CT) with intravenous contrast was performed, and at CT, a severe dilation of the common biliary duct was detected. The common biliarytract was grossly interrupted at the terminal end by compression of the papillary area due to the neoplastic infiltration of the stent mesh (Figure 2A). After multidisciplinary consultation, because of the impossibility of reaching the papilla endoscopically due to tumor growth through duodenal stent’s mesh (Figure 3), an endoscopic–radiological rendezvous approach was proposed. A percutaneous transhepatic cholangiography (PTC) was performed and a hydrophilic guidewire (0.35″, 260cm, Merit Medical, South Jordan, UT, USA) was introduced through a catheter (MPA, Beacon Tip, 5F, Cook Medical, Bloomingtom, IN, USA) into the main biliary branch in order to reach the major papilla, but it was not possible due to neoplastic infiltration of the metal mesh of the stent and the presence of contextual fibrosis (Figure 4). A Teflon-coated guide (V18 Controlwire, 0.018″, 182 cm, Boston Scientific, Marlborough, MA, USA) was introduced and it was possible to penetrate between the stent mesh and reach the duodenal lumen (Figure 5).Subsequently, a wire-guided sphincterotome (Clevercut 3V, Olympus, Tokyo, Japan) was pushed up into the papilla and 0.035″ guidewire (Visiglide, Olympus, Tokyo, Japan)was inserted through the sphincterotome into the common bile duct; the guidewire was promptly engaged by a retrieval device (One-snare, 5F, Merit Medical, South Jordan, UT, USA) inserted into a percutaneous catheter and pulled outside through it (Figure 6). Aballoon (WANTY, 5 × 80 mm, Barty Medical, Hangzhou, China) was loaded onto the 0.035″ guidewire and positioned between the stent mesh and the major papilla: it was inflated to create a “crash stent action”, performed to facilitate the passage of the stent in the main biliary tract(Figure 7 and Figure 8). Using the same percutaneous access, a plastic biliary prosthesis was inserted (9 cm, 10 Fr, Cotton-Huibregtse plastic biliary stent, Cook Medical, Limerick, Ireland), resolving both bile duct and duodenal obstructions (Figure 9). The two interventions were performed under general anesthesia in the same session in order to ensure stable conditions. Clinical symptoms rapidly improved, and the patient was able to take solid food with the resolution of symptoms. Unenhanced CT was performed in order to assess the correct positioning of the stent (Figure 2B). She was discharged 2 days after the procedure.

## 3. Discussion

Duodenal metastases by cervicalcancer are extremely uncommon;only sixcases are described in the literature [1,2,11,12]. The prognosis is poor, and patients often present an impaired performance status, accompanied by substantial weight loss and a state of malnutrition that can contribute to morbidity related to the disease and to the ability to undergo therapeutic intervention. In this setting, symptom palliation and maintenance of an acceptable quality of life are of crucial importance. The surgical approach of gastrojejunostomy has been considered the gold standard for palliation, but in the last decade, endoscopic stenting with self-expandable metallic stents (SEMSs) has emerged as a mainstay treatment compared with surgery [5,7,13,14,15,16,17]. Dormian et al. [18] reported that SEMSs is technically successful in 97% of cases and clinically successful, measuredas the ability of a patient to tolerate oral intake, in 87%. However, SEMSs are associated inthe long term witha high risk of stent occlusion and migration;other complications reported are duodenal bleeding and perforation [19]. SEMSs should probably be considered the best option in patients with poor general condition and a theoretically short life expectancy;meanwhile, surgery still represents a first optionfor patients with a longer life expectancy. Here, a plastic biliary prosthesiswas placedbecause of the shorter life expectancy;in fact, the patient died 3 months after the procedure. The combined biliary and gastric outlet obstruction was described by the Mutignani et al. classification system [20], based on the anatomical location of the duodenal stricture in relation to the papilla. According to the classification system, this case was a type II stenosis because of the involvement of the second part of the duodenum and papilla. In this case, anendoscopic procedure alonewould have beendifficult, so a biliary stenting through the mesh ofthe duodenal SEMS was used to resolve the double strictures using a procedure known as the rendezvous technique, an intervention thatcombines both endoscopic and percutaneous approaches [18,19].

## 4. Conclusions

The presented rendezvous technique offers a minimally invasive alternative to surgery in patients with both biliary and duodenal obstruction and short life expectancy, resulting in with symptom relief and improvedquality of life, although this procedure must not be considered curative.

## Data Availability

This article did not report any data, apart from those reported here.
